# Supplementary material for: Daily rhythm in cortical chloride homeostasis underpins functional changes in visual cortex excitability
Source: Nat Commun. 2023 Nov 4;14:7108. doi: 10.1038/s41467-023-42711-7 (PMC10625537; doi:10.1038/s41467-023-42711-7)
Supplement: Supplementary file 1 — Supplementary Information [file 41467_2023_42711_MOESM1_ESM.pdf]

Daily rhythm in cortical chloride homeostasis underpins functional changes in visual cortex excitability

E. Pracucci, R. Graham, L. Alberio, G. Nardi, O. Cozzolino, V. Pillai, G. Pasquini, L. Saieva, D. Walsh, S. Landi, J. Zhang, A.J. Trevelyan, G.M. Ratto

Supplementary Figures

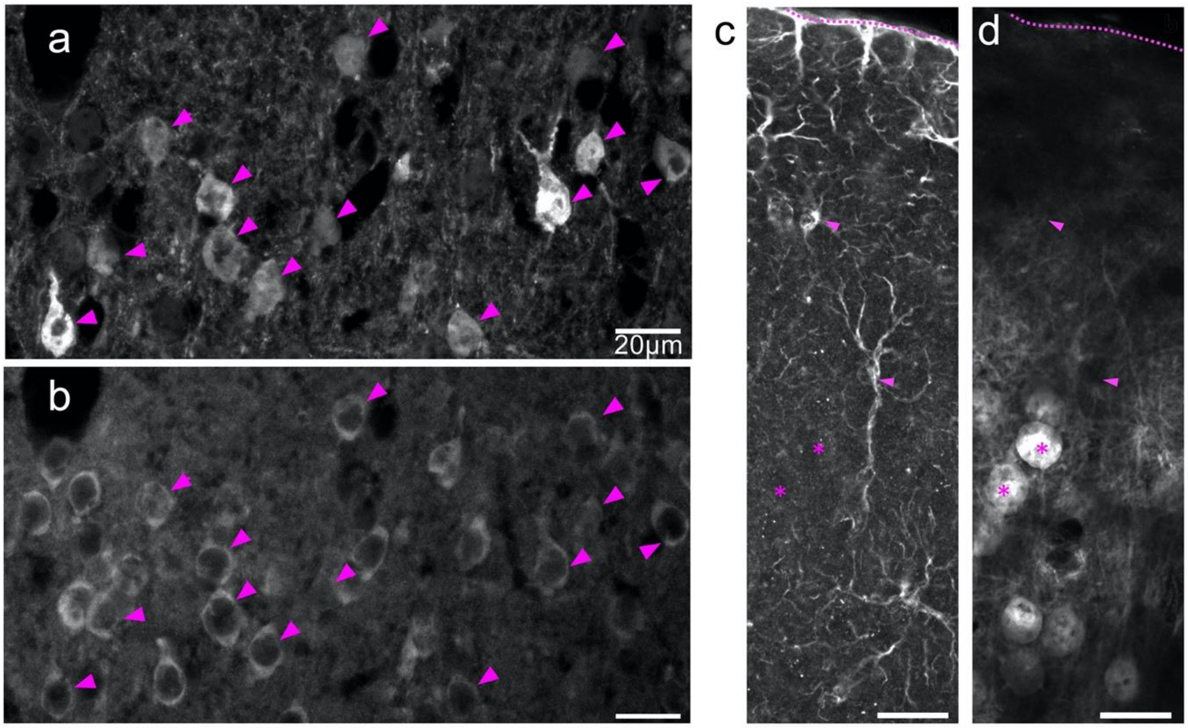

Supplementary figure S1. LSSmClopHensor labelling is restricted to pyramidal cells.

a) GFP / LSSmClopHensor labelling and **b**) CamKII immunoreactivity in supragranular neocortex. Note that LSSmClopHensor co-labels with staining of pyramidal cells. **c**) GFAP immunoreactivity and **d**) GFP / LSSmClopHensor labelling in another neocortical brain slice. Arrowheads indicate prominent glial processes, and the stars are locations of LSSmClopHensor labelled cell bodies. Note the absence of LSSmClopHensor in the glial cells. This is consistent with previous reports showing that Emx1-dependent expression labels pyramidal cells and not glia, in adult mice (Gorski et al., 2002; Parrish et al., 2023). Scale bar=20 µm in all panels.

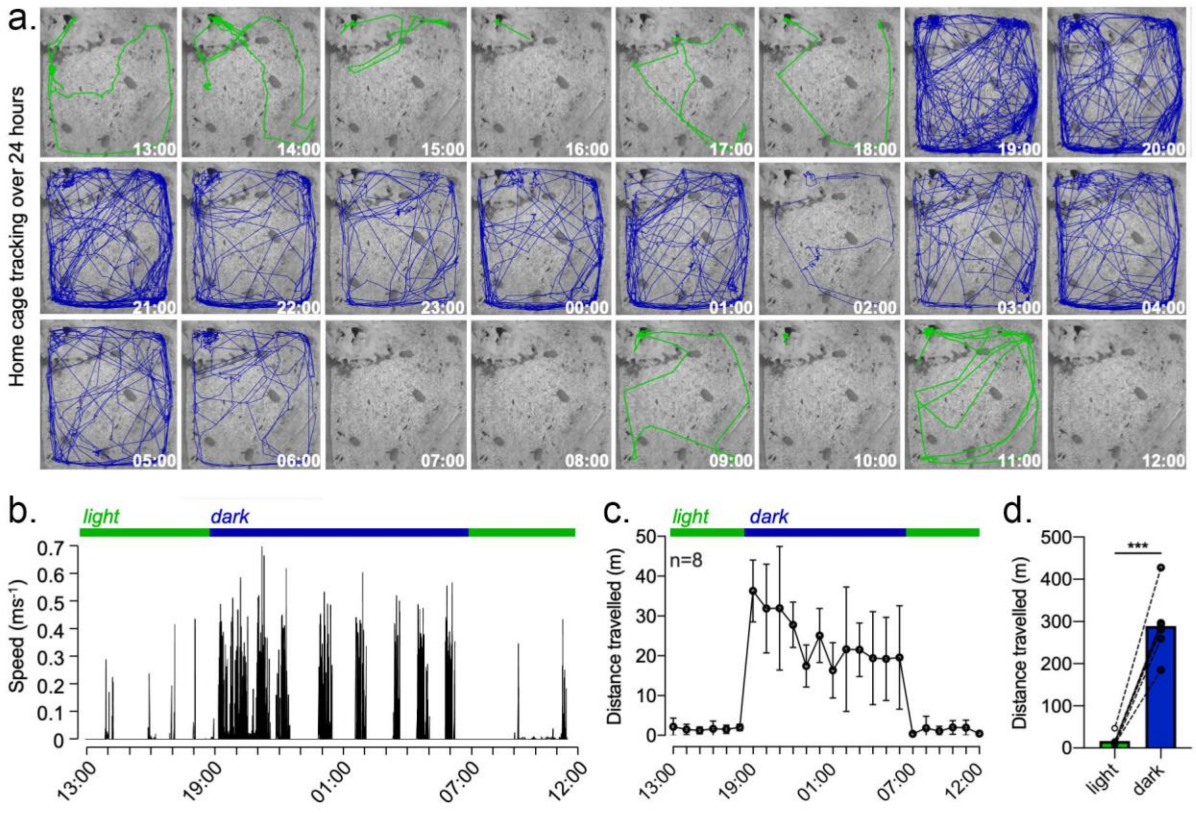

Supplementary figure S2. Mice are active almost exclusively during periods of darkness.

a) Each panel shows the movement of a single mouse during a single hour, as specified, sampled every 2 s. Green lines show activity during the light periods (ZT 5-ZT 17), and blue lines during the dark periods (ZT 17-ZT 5). **b**) A higher resolution depiction of the movement speed for a single mouse. Note the sudden transformation of activity, almost as soon as the lights are turned off, and while there are subsequently periods of inactivity during nighttime, the activity remains far higher than during the day, when mice are almost entirely inactive. **c**, **d**) The pooled data (n=8 mice) show that this pattern was repeated in all mice and is in line with the known behaviour patterns of mice in the wild. Source data are provided in the Source Data file.

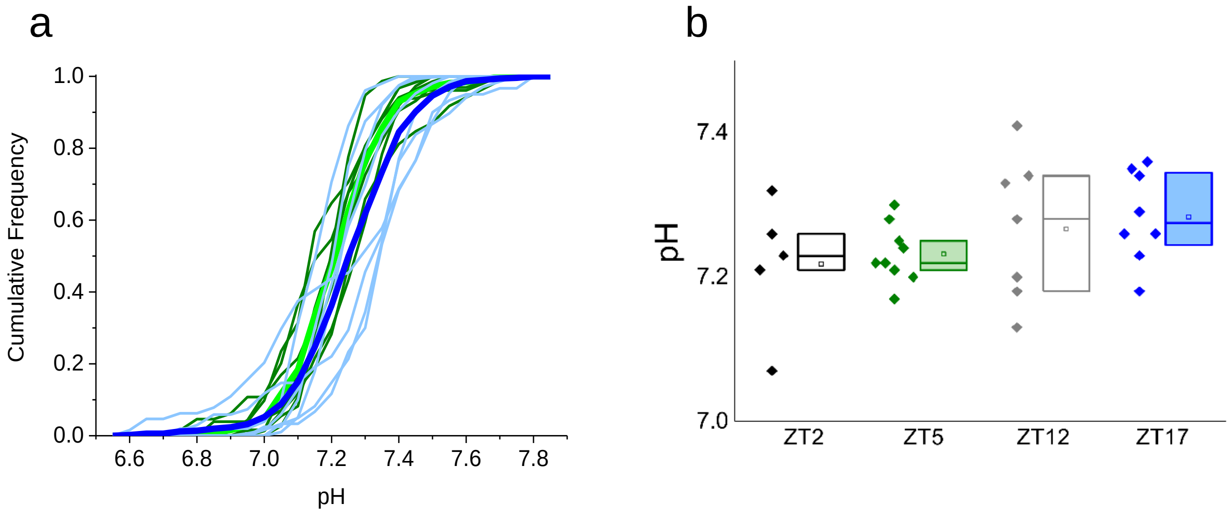

Supplementary figure S3. Intracellular pH, imaged using LSSmClopHensor, shows negligible changes over the course of the day.

a) Cumulative distribution for each mouse imaged at ZT 5 (green traces) and at ZT 17 (blue traces). The thick lines show the mean distributions of these two groups. **b**) Median pH values for each individual mouse, grouped by the four different times they were imaged (ZT 2, 9 am; ZT 5, midday; ZT 12, 7 pm; ZT 17, midnight). A one-way ANOVA revealed that there was no statistically significant difference in median pH values between the different imaging time groups ($F_{3, 25}$=[1.14], p=0.35). Source data are provided in the Source Data file.

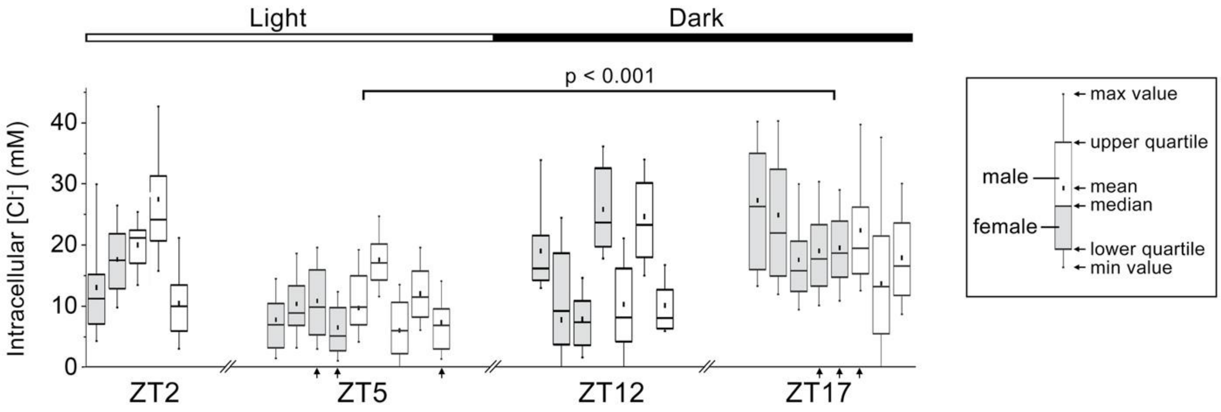

Supplementary figure S4. Distributions of $[Cl^-]_i$ for every mouse.

Distribution of values from all 29 mice that were imaged. For each animal, we show sex of the animal (female – gray; male – white), the full range, mean, median and interquartile range, as indicated in the inset panel. We have grouped the data into the 4 sets of times (ZT 2, 9 am; ZT 5, midday; ZT 12, 7 pm; ZT 17, midnight; daylight for the first two, and night-time for the last two); within each group, the data is ordered by the experimental index of the animal. A one-way ANOVA revealed that there was a statistically significant difference between at least two of the experimental groups ($F_{3,\,25}$=[5.2], p=0.0063); this was between the medians of the midday (ZT 5) and midnight (ZT 17) data (p=5.7 x 10^{-4}, Mann-Whitney), while all other comparisons were not significantly different.

Arrowheads on the abscissa indicate those animals studied in Newcastle, including 3 animals at ZT 5 (2 F, 1 M) and 3 at ZT 17 (2 F, 1 M). Note that the Pisa (no arrows) and Newcastle (arrowed) data align well, and both show clear day to night modulation. Linear regression on the ZT 5 and ZT 12 subset (the only ones where there is both Newcastle and Pisa data) indicated that the day-night difference was highly significant (p=0.00012) and the Pisa-Newcastle distinction was not (p=0.26). The R^2 term was very similar whether or not the location was included in the analysis (with both, R^2=0.67; omitting location, R^2=0.64). Likewise, similar analysis of sex differences revealed no significant difference between male / female animals (p=0.18; including both sex / time of day, R^2=0.68; omitting location, R^2=0.64). Source data are provided in the Source Data file.

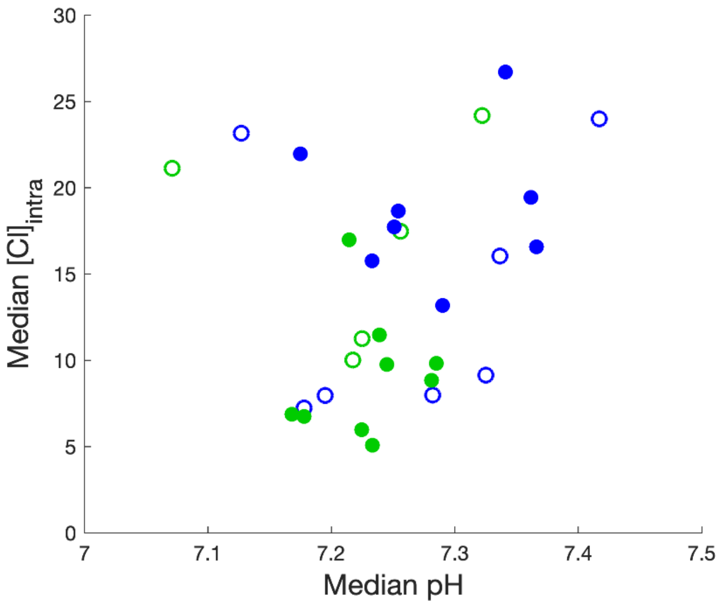

Supplementary figure S5. Median pH versus $[Cl^-]_i$ for every mouse.

We observed no systematic relationship between the median values of pH and $[Cl^-]_i$ across the entire set of imaged mice ($p=0.22$, $n=29$ paired data points). Green data points are from mice imaged in the day (open symbols, ZT 2 (9 am); filled symbols, ZT 5 (midday)) and blue data points are from mice imaged at night (open symbols, ZT 12 (7 pm); filled symbols, ZT 17 (midnight)).

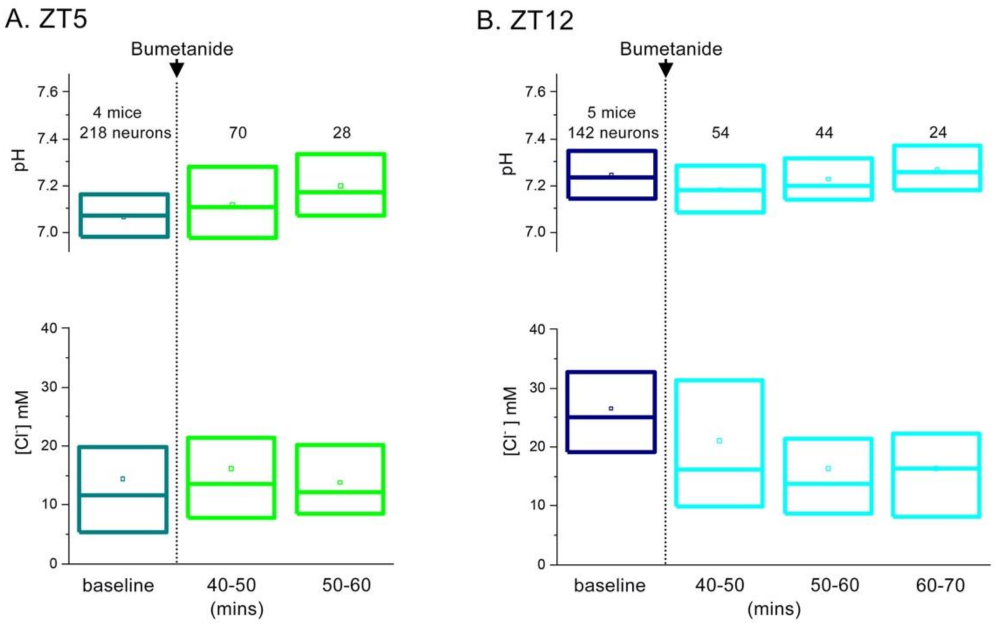

Supplementary figure S6. Time course of effect of bumetanide application on $[Cl^-]_i$.

Quantification of intracellular pH and $[Cl^-]$ before and after bumetanide application either at ZT 5 (**A**) or ZT 17 (B). In each case we show the 1st and 3rd quartile, median (central line) and mean (spot) for each experimental group, with the sample size indicated above the pH plots. A Kruskal-Wallis one-way Analysis of Variance on Ranks revealed no statistical differences in pH after bumetanide application at either time of day, or in the effect on $[Cl^-]_i$ at ZT 5. In contrast, at ZT 17, there was a highly significant drop in $[Cl^-]_i$ after bumetanide application (Kruskal-Wallis, $p=<0.001$). The pharmacological effect at ZT17 appeared to be complete by about 50 mins. Source data are provided in the Source Data file.

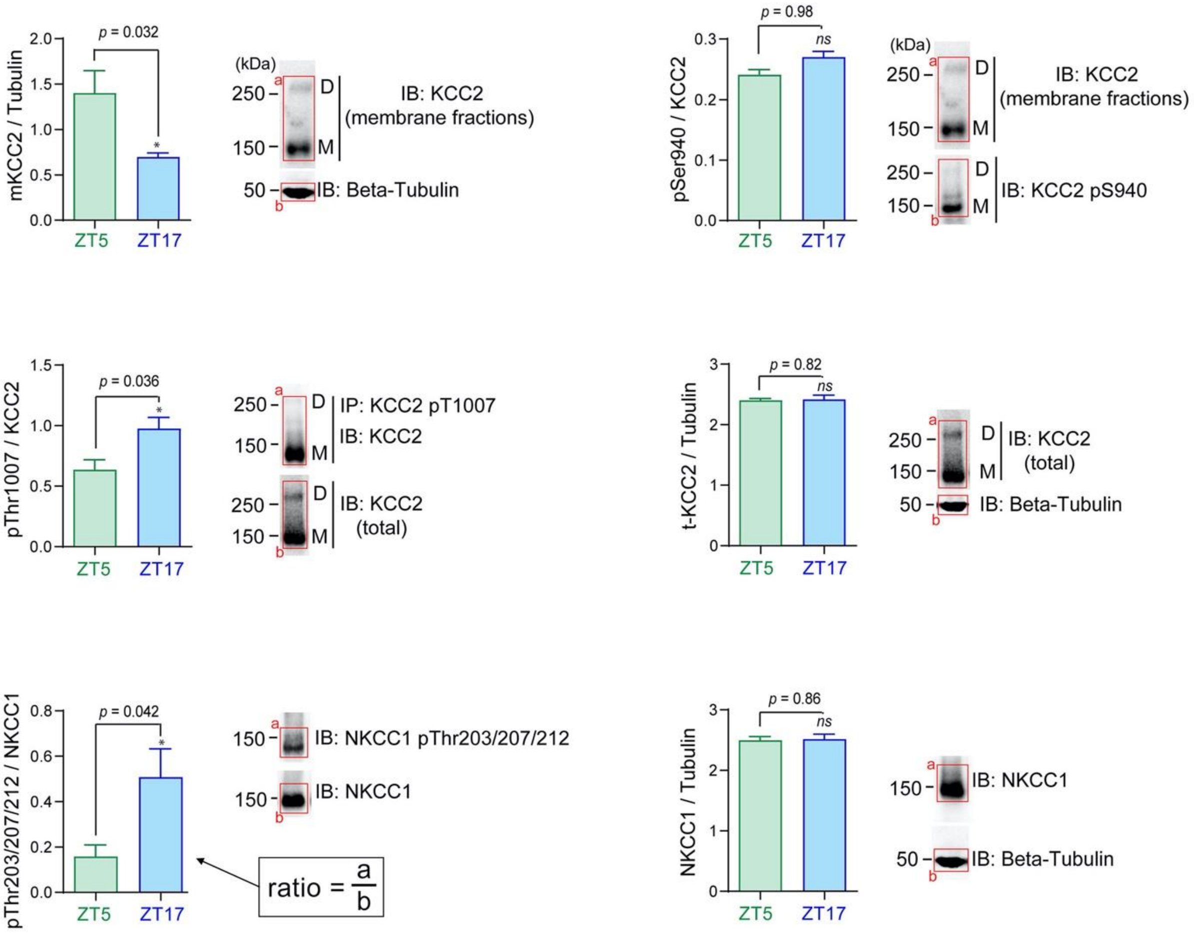

Supplementary figure S7. Analysis of western blot labelling.

The ratios for the 6 panels in Figure 3 are calculated as the ratio of the intensity of the bands outlined by box a, divided by that in box b. Source data are provided in the Source Data file.

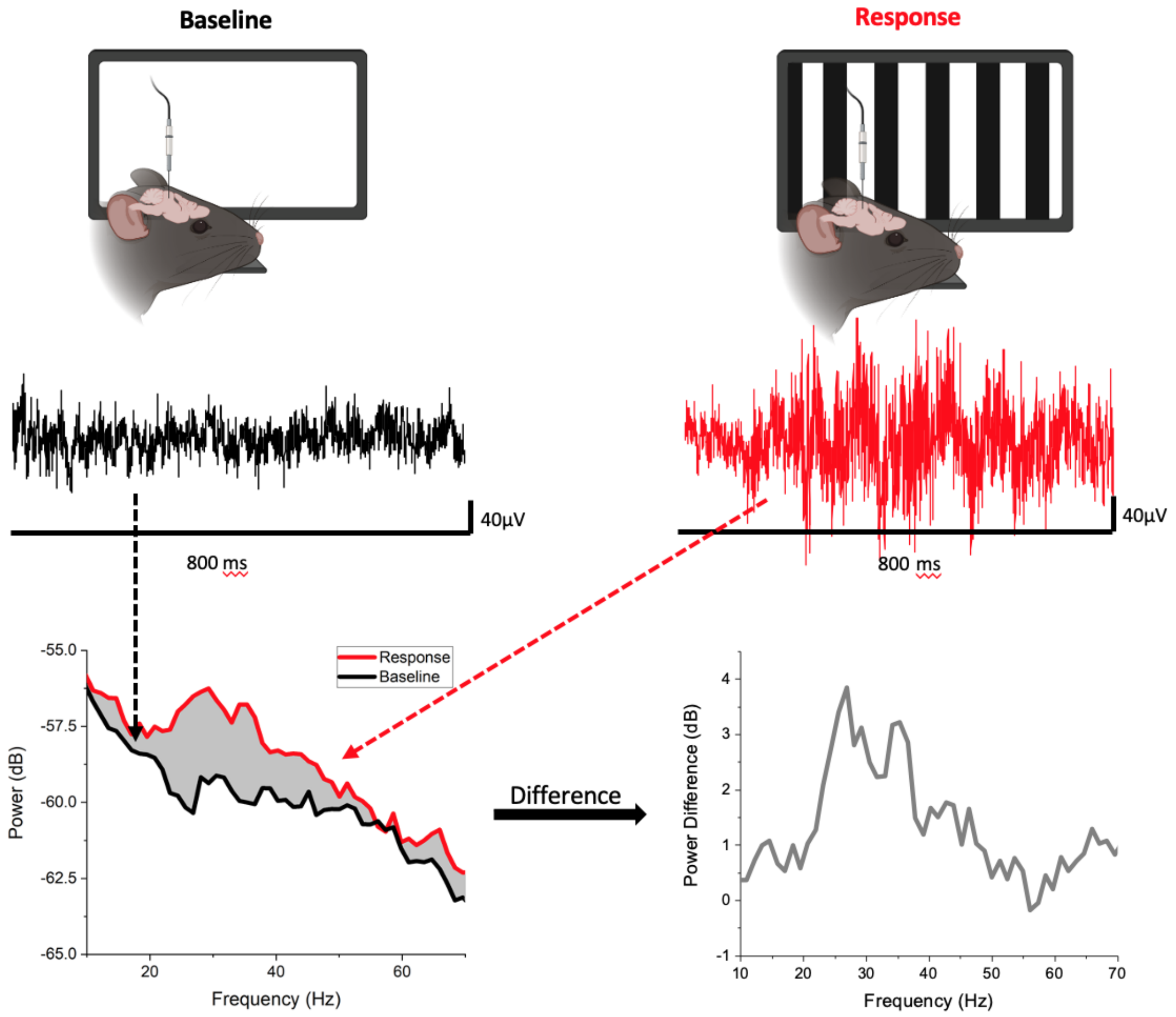

Supplementary figure S8. Schematic to illustrate the frequency analyses of the visual response assay (figure 4).

Electrode arrays were implanted chronically in the visual cortex, and the animals were allowed to recover before being trained to attend to visual presentations on a screen. Short presentations of visual gratings (2 s) were given, and Fourier analysis was performed on the recorded local field potential, comparing a short baseline epoch (black) immediately prior to the visual presentation, and the activity during the presentation (red). The difference between these is what is presented in Figure 4.

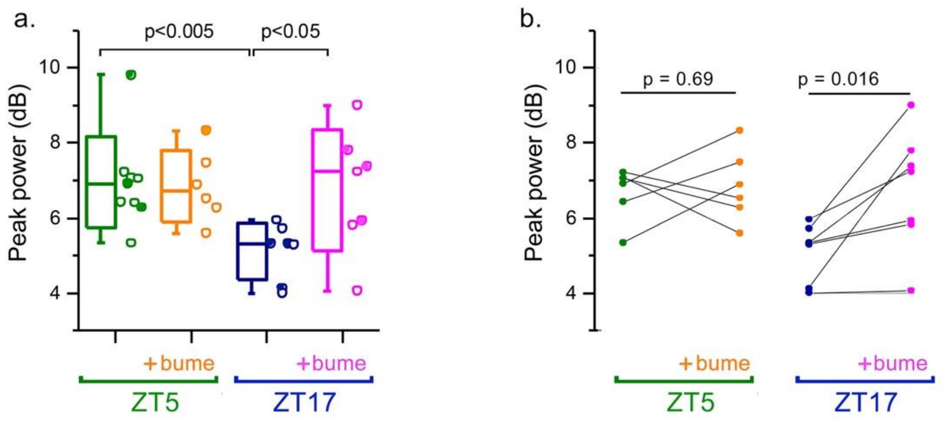

Supplementary figure S9. Paired data points showing the effect of bumetanide on visual responses, for each animal, as shown in Figure 5.

a) A reproduction of Figure 5d is reported here. **b**) Data obtained from the same subject before and after bumetanide superfusion are connected with a line. P-values of paired sample Wilcoxon signed rank tests are shown. Source data are provided in the Source Data file.

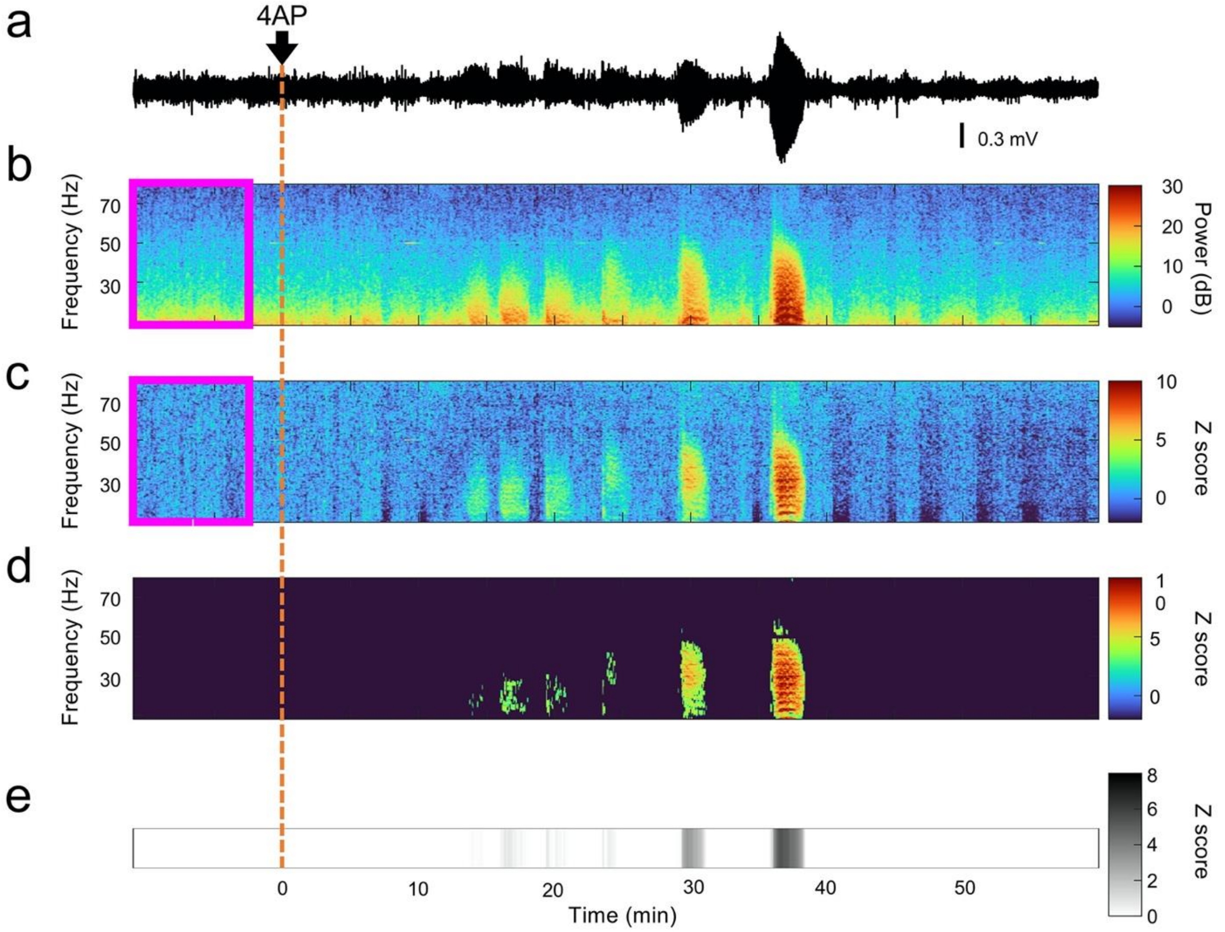

Supplementary figure S10. Computation of the z-scoring of epileptiform activity.

a) An example trace of the LFP recording, during a midnight 4-AP experiment. 4AP was microinjected over a period of 5 min, starting at time 0. **b**) spectrogram of the same trace (computed using the Matlab function *mtspecgramc.m*; time windows of 10 s with steps of 1 s, 5 tapers and time-bandwidth product=3). Frequencies between 8 Hz and 100 Hz were used. Note that power attenuates at high frequencies. The magenta frame highlights a baseline period before 4AP injection. **c**) Z-transform of the spectrogram in b computed as follows: for each frequency f, we computed the mean (μ_f) and the standard deviation (σ_f) of the power during the baseline. Then, for every element of the spectrogram x_{ft}, the z transform was obtained with $(x_{ft} - \mu_f) / \sigma_f$. Note that this normalization overcomes the attenuation of the power at high frequencies. **d)** a binary mask was applied to the z-transform of the spectrogram to keep only values higher than 4 (4 σ higher than the baseline power). **e**) the z-score time course was computed, summing the z-transform of the spectrogram across all frequencies (columns). Source data are provided in the Source Data file.

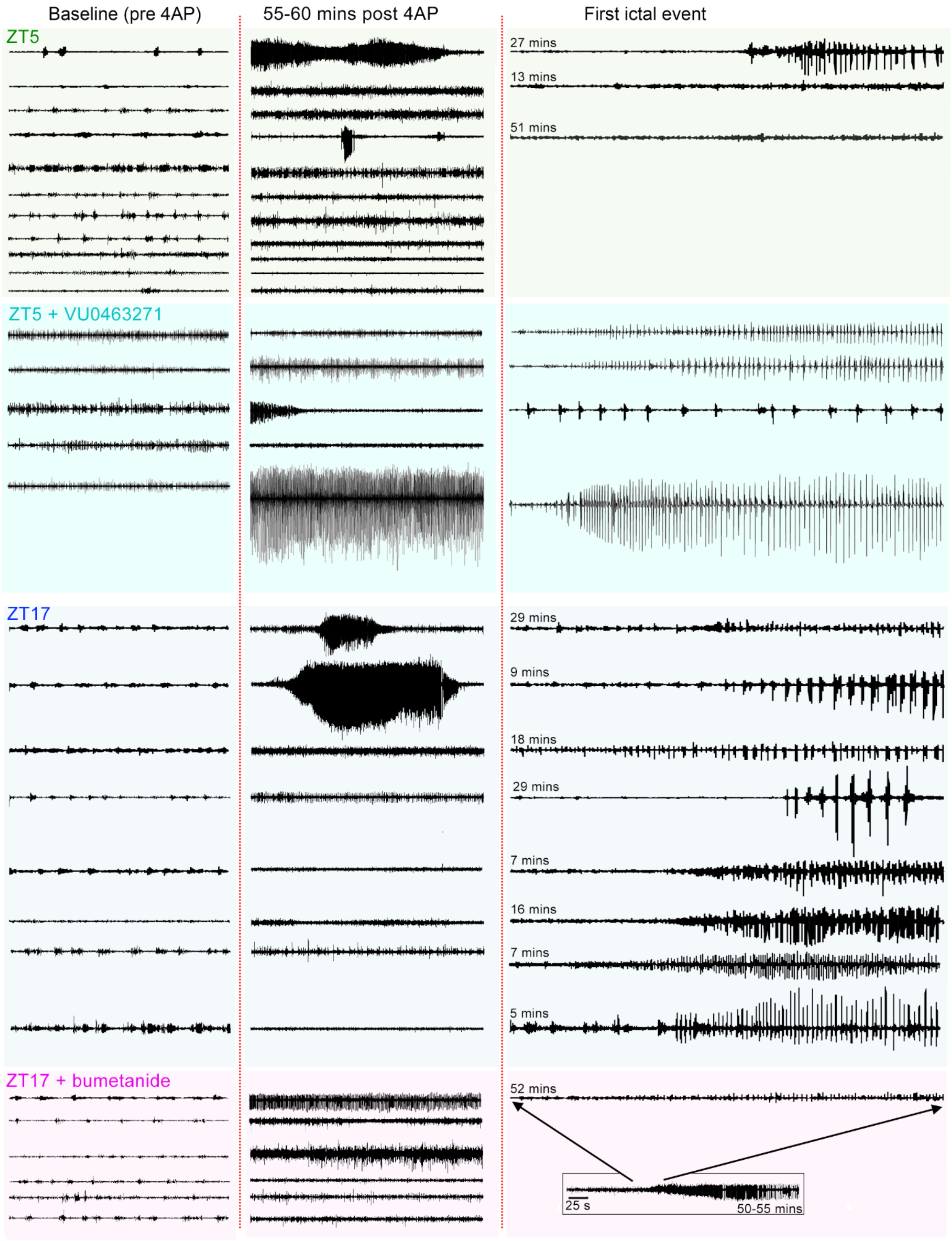
Baseline (pre 4AP)
55-60 mins post 4AP
First ictal event
ZT5
27 mins
13 mins
51 mins
ZT5 + VU0463271
ZT17
29 mins
9 mins
18 mins
29 mins
7 mins
16 mins
7 mins
5 mins
ZT17 + bumetanide
52 mins
25 s
50-55 mins

Supplementary figure S11. Example segments of every 4AP recording.

In each case we show the baseline activity prior to 4-AP application (left) and the activity during the final 5 minutes of the recordings (middle). We also show a zoomed-in, 30s epoch at the start of the first seizure, in every animal that progressed to having a seizure within 1 hour of 4-AP application. Note that in instances where the seizure evolved very slowly (see for example the ZT 17 + bumetanide example, which is shown at low resolution in the inset), the high-resolution image may only capture the very early stages and so are not so striking (other instances of this are seen in the ZT 5 examples). All ZT 17 experiments (8/8) and 4/5 experiments at ZT 5 with VU0463271 pre-treatment progressed to show full ictal activity, whereas only 3/11 of the ZT 5, and 1/6 of the experiments at ZT 17 with bumetanide pre-treatment, progressed to seizures. Data were bandpass filtered in the 8-80 Hz band. Source data are provided in the Source Data file.
